# Chromogenic bacterial staining of teeth: a scoping review

**DOI:** 10.1186/s12903-025-05441-4

**Published:** 2025-01-11

**Authors:** Mathangi Kumar, Medhini Madi, Ravindranath Vineetha, Divya Gopinath

**Affiliations:** 1 Department of Oral Medicine and Radiology, Manipal College of Dental Sciences, Manipal, Manipal Academy of Higher Education, Manipal, 576104 Karnataka India; 2 Basic Medical and Dental Sciences Department, College of Dentistry, Ajman University, Ajman, UAE

**Keywords:** Bacteria, Chromogenic, Staining of teeth, Aesthetics, Scaling

## Abstract

**Background:**

The purpose of this scoping review is to understand the etiological, clinical characteristics and treatment of chromogenic staining of teeth and the various management strategies reported in literature. This SR was performed in accordance with the PRISMA 2022 guidelines and was registered in the PROSPERO database (CRD42024565446).

**Methods:**

A systematic electronic search on databases like Scopus, Medline, EMBASE, CINAHL, ProQuest and Web of Science from inception to July 2024 was performed. Two independent reviewers ran the search strategy in the mentioned databases.

**Results:**

A total of 989 articles were obtained from various databases. 21 were included for data extraction of which 2 were case reports, 1 was case series and 18 were research articles. The prevalence of staining due to chromogenic bacteria reported from the included studies ranged from 3.1 to 18.5%. All these studies reported on the typical black color of staining of teeth.

**Conclusion:**

This scoping review reveals sparsity in existing literature regarding the etiological, clinical characteristics and management of chromogenic staining of teeth. Though the role of peculiar oral microbiota is well established, evidence regarding the management strategies to combat these recalcitrant staining remains a strong research question.

**Supplementary Information:**

The online version contains supplementary material available at 10.1186/s12903-025-05441-4.

## Background

Pigmented deposits on the surfaces of the teeth are termed as stains. Generalized staining of teeth is a common dental complaint encountered in clinical practice. It poses aesthetic concerns and is the most common reason for which patients report for dental treatment. Staining of teeth may be caused due to various intrinsic and extrinsic factors [1]. Intrinsic staining of teeth refers to endogenous factors that lead to discoloration of teeth. The causes for intrinsic teeth discoloration may be due to developmental disorders like amelogenesis and dentinogenesis imperfecta. Dental fluorosis, trauma and certain medications(tetracycline, minocycline) also result in intrinsic coloration of the teeth [2, 3].

Extrinsic staining of teeth arises due to the accumulation of exogenous pigments. This may be due to the use of tobacco products (smoking and smokeless forms), food and beverages (tea, coffee etc.), restorative materials (amalgam), medications (iron, iodine, herbal preparations etc.) [2, 3]. The type of extrinsic stains depends on the type of causative agent. Brown stains are thin, acquired pellicle on the surfaces of teeth mostly due to insufficient brushing and tannins. Dark brown or black surface deposits with brown discoloration of teeth is typically seen in extrinsic staining caused due to tobacco products. Tobacco stains cause tough tenacious brown surfaces of teeth due to the release of combustion products of smoked form of tobacco which attaches to the pre-existing pellicle on the surface of teeth. Further, smokeless form of tobacco results in the release of tobacco juices into the irregularities of teeth resulting in staining [4]. Green staining of teeth has been associated with organisms like Aspergillus and Pencillium. Orange stains are predominantly attributed to Serratia marcescens and Flavobacterium lutescens [2].

**Fig. 1:**
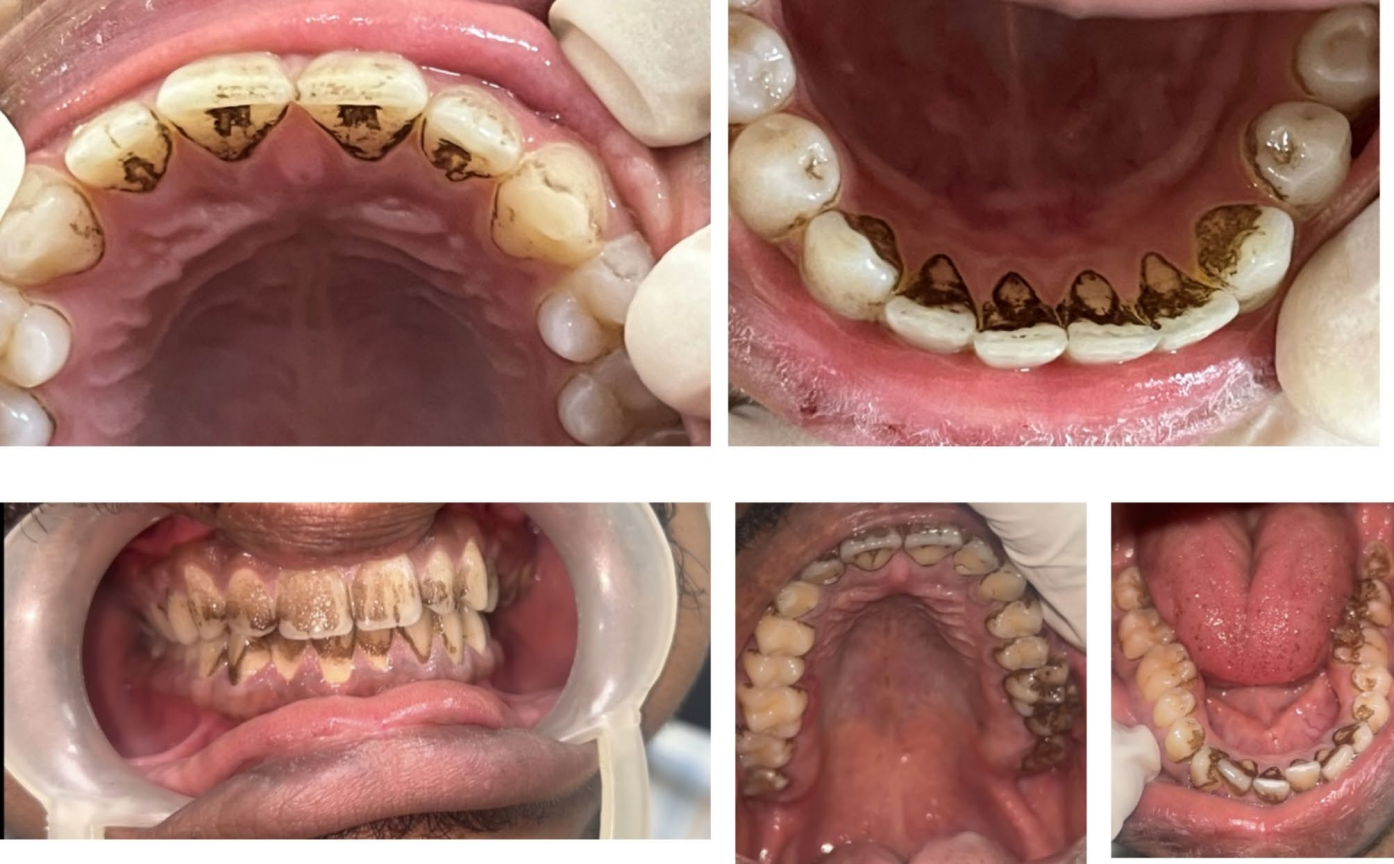
Clinical photographs of patients with chromogenic staining of anterior and posterior teeth

Chromogenic staining is a type of extrinsic staining of teeth caused by certain color producing bacteria. Bacterial organisms cause a variety of color changes on the teeth surfaces ranging from green, black, brown to orange. These stains have been reported to affect primary dentition and permanent dentition [5–7]. These color changes and patterns in clinical presentations are peculiar in case of chromogenic staining (Fig. 1). These stains are noted along the cervical thirds of the teeth in the form of confluent dots. In routine clinical practice, it has been found that they are resistant to the conventional treatment modalities. They reappear to cause staining in a span of weeks to months [8]. Hence this scoping review aimed to explore the various etiological, clinical characteristics and treatment of chromogenic staining of teeth and the various management strategies reported in the literature.

## Methods

### Study design

This scoping review was conducted using the PRISMA (Preferred Reporting Items for Systematic Reviews and Meta-analyses) Extension for Scoping Reviews [9]. This project was registered in PROSPERO (CRD42024565446).

### Search strategy

A systematic electronic search on databases like Scopus, Medline, EMBASE, CINAHL, ProQuest and Web of Science from inception to July 2024 was performed. Two independent reviewers ran the following search strategy in the mentioned databases. The search strategy included the following terms: chromogenic AND bacteria* AND teeth AND staining, chromogenic AND stains AND teeth*, Black stain AND chromogenic AND dentition. The relevant studies were also manually searched to avoid missing out on suitable references. The grey literature on this topic was searched in relevant subject specialty textbooks and Google scholar to ensure a comprehensive search. The two authors independently performed the title and abstract screening (MK, MM). Any conflicts that arose were resolved by the third reviewer (RV). Authors MK and MM independently reviewed all the articles that met the inclusion criteria for full-text screening. A detailed tabulation from various databases and the filters applied is depicted in Supplementary material 1.

### Inclusion criteria and exclusion criteria

All clinical trials, cross sectional studies, observational studies, case reports, case series in English language evaluating the clinical characteristics and management strategies of chromogenic staining in humans (children, adults and geriatric involving both genders) were included. Comprehensive data regarding the clinical and management aspects of chromogenic staining of teeth were documented. All animal studies, letters to editors, commentary, narrative, scoping, systematic review articles, and dissertations were excluded. Studies and reports addressing staining of teeth caused due to other reasons (other than chromogenic organisms) were excluded.

### Screening and data extraction

Two reviewers independently performed the data extraction using a pre-designed pilot tested data extraction sheet. Any disagreements during the extraction process were resolved by discussion between the reviewers. In case of unpublished and missing information/data, the study authors contacted the librarians and requested for the full text. All the data collected was switched among reviewers to check for any discrepancies. The relevant data were analyzed from all the articles that met the inclusion criteria and the information was systematically recorded as depicted in Table 1.

**Table 1:** Study characteristics from the included research articles on chromogenic staining of teeth

S. No.	Author, Year, Country	Age (Years)	Type of literature	Dentition	Prevalence	Sample size	Color	Organism
1.	Elelmi Y et al., 2020 (Tunisia)	4–5	Research (Descriptive cross-sectional study)	Primary	24 (6.1%)	393	Black	NS
2.	Mutsaddi S et al., 2018 (India)	3–11	Research (Case control study)	Mixed	30	30	Black	A.naeslundii, A. actinomycetemcomitans, F. nucleatum
3.	Li Y et al., 2015 (China)	4–5	Research (Analytical study)	Primary	25	25	Black	NS
4.	Akyüz S et al., 2015 (Turkey)	5–13	Research (Cross-sectional study)	Mixed	60 (18.5%)	325	Black	NS
5.	Gayatri A et al., 2017 (Indonesia)	4–11	Invitro laboratory experiment	Mixed	NS	NS	Black	Actinomyces spp, Prevotella spp
6.	Martin JMG et al., 2013 (Spain)	6	Epidemiological	Primary	3.10%	3272	Black	NS
7.	Dwiputri E et al., 2018 (Indonesia)	4–8	Research (Cross-sectional study)	Mixed	21 (6%)	378	Black	NS
8.	Zhang Y et al., 2022 (China)	3–4	Research (Cross-sectional study)	Primary	31 (12.4%)	250	Black	Neisseria, Lautropia, Haemophilus, Aggregatibacter
9.	Zhang F et al., 2017 (China)	3–6	Research (Experimental pilot study)	Primary	NS	10	Black	Actinomyces
10.	Veses V et al., 2020 (Spain)	43.8 ± 15.8	Research (Analytical study)	Permanent	NS	18	Black	Bacteroidetes, Actinobacteria
11.	Chen L et al., 2019 (China)	4.34 ± 0.81	Research (Cross-sectional study)	Primary	NS	100	Black	Firmicutes, Fusobacteria, Proteobacteria, Bacteroidetes, Actinobacteria, Candidate_division_TM7
12.	Çelik ZC et al., 2021 (Turkey)	26.4 ± 8.4	Research (Analytical study)	Permanent	NS	26	Black	Aggregatibacter, Bergeriella, Brachymonas, Capnocytophaga, Corynebacterium, Neisseria, Rothia, Streptococcus
13.	Yanhui L et al., 2016 (China)	NS	Research (Analytical study)	Primary	NS	65	Black	Leptotrichia and Fusobacterium for blast staining; Streptococcus and Mogibacterium for black staining and caries mixed; unclassified Clostridiaceae, Peptostreptococcus, and Clostridium for mixed
14.	Heinrich-Weltzien R et al., 2014 (Germany)	7.9 ± 1.3	Research (Case control study)	Mixed	113 children (1.5%) among the total population of 7,624 children	46	Black	S. mutans, S. sobrinus, Lactobacillus sp., A. naeslundii, P. gingivalis, A. actinomycetem, P. intermedia, F. nucleatum
15.	Ortiz-López CS et, 2018 (Spain)	39.9 ± 18.3	Research (Case-control study)	Permanent	NS	47	Black	NS
16.	Carelli et al., 2024 (Italy)	22	Research (Case-control pilot study)	Permanent	NS	16	Black	P. intermedia, A. actinomycetemcomitans, Veillonella spp. Actinomyces spp.
17.	Lihala R et al., 2019 (India)	18–40	Research (Experimental study)	Permanent	NS	50	Black	grampositive rods, Actinomyces
18.	Lavine P et al., 2018 (Indonesia)	4–11	Research (Invitro laboratory experiment)	Mixed	NS	3	Black	Actinomyces spp.
19.	Yamada M et al., 2024 (Japan)	3	Case series	Primary	2	NA	Black	NS
20.	Bussell RM et al., 2010 (United Kingdom)	4	Case report	Primary	1	NA	Navy blue	Pseudomonas aeruginosa
21.	Alammari ST et al., 2024 (Saudi Arabia)	11	Case report	Mixed	1	NA	Black	NS

NA: Not Applicable; NS: Not specified

## Results

### Study selection

**Fig. 2:**
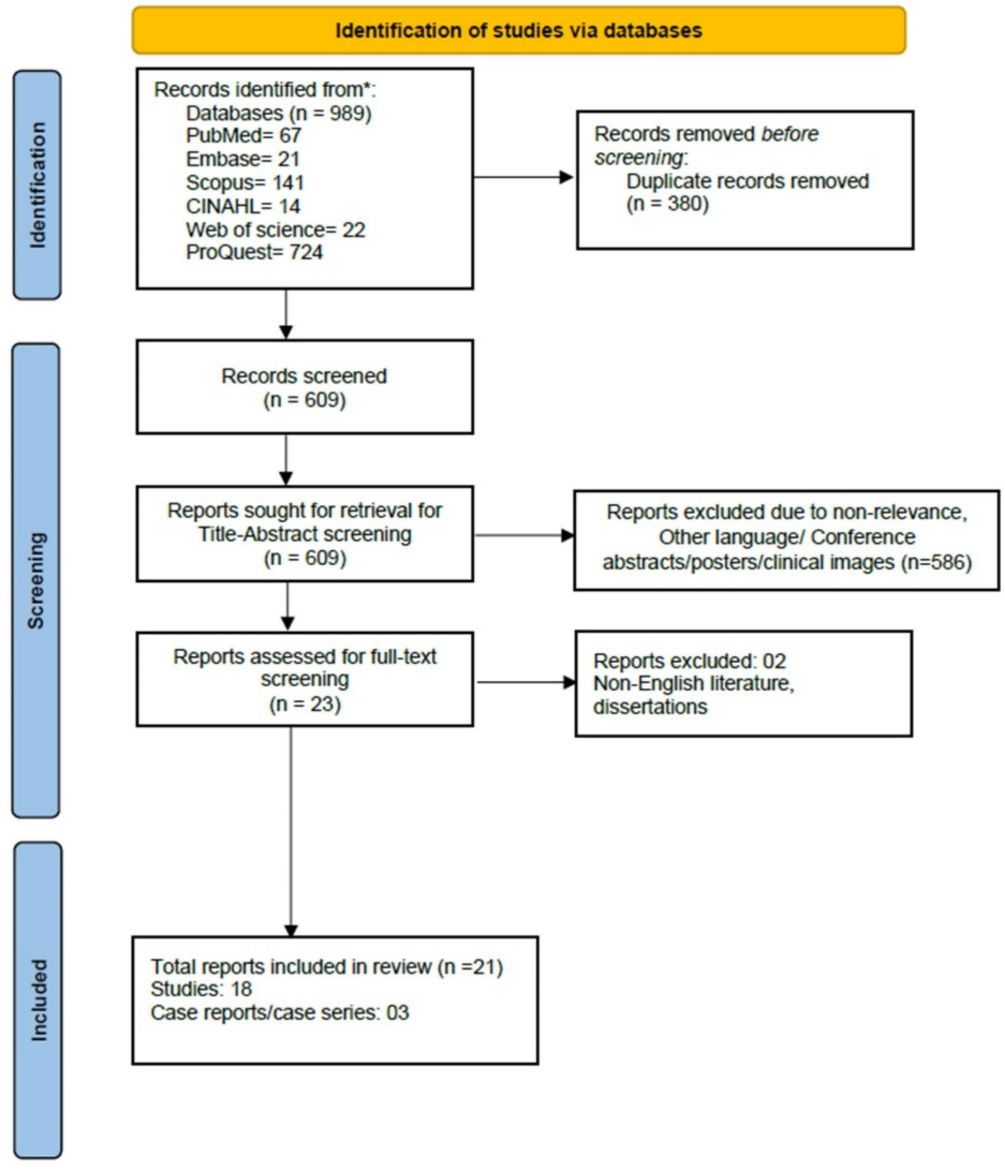
PRISMA flowchart depicting the selection of included articles

The flowchart of the literature screening process is shown in Fig. 2. A total of 989 articles were obtained from various databases. 389 articles were duplicates which were detected via Rayyan software. 609 articles that were retrieved were screened for title and abstract. 586 of them were excluded due to non-relevance, non-English literature, conference abstracts and clinical images. 23 articles were screened for full text of which 21 were included for data extraction.

### Study characteristics

21 articles were included of which 2 were case reports [8, 10], 1 was case series [11], descriptive cross sectional studies [12–16], case control study [6, 17–19], analytical study [7, 20–22], invitro laboratory experiment [23, 24], epidemiological [25], experimental study [26, 27]. The complete list of included studies are presented in Table 1.

### Case reports and case series

Literature report on a 4-year-old child from United Kingdom with West syndrome reported navy blue staining of teeth with Pseudomonas aeruginosa being the causative organism isolated. In this case, there were multiple drugs implicated (lamotrigine, topiramate, clonazepam, midazolam, baclofen) [10]. Black stain was the commonly occurring color of teeth staining in a case report and case series by Sattam T. Alammari [8] and Yamada M et al. [11] The staining of teeth reported in the above mentioned cases were resistant to regular toothbrushing and conventional scaling of teeth.

### Research articles

Analysis of staining of teeth due to chromogenic bacteria was performed in primary dentition [7, 12, 15, 16, 20, 25, 26], mixed dentition [13, 14, 17, 18, 23, 24] and permanent dentition [6, 19, 21, 22, 27]. The prevalence of staining due to chromogenic bacteria reported from the included studies ranged from 3.1 to 18.5%. All these studies reported on the typical black color of staining of teeth. The microorganisms isolated in these studies that are thought to be implicated in the causation of chromogenic staining along with their characteristics are depicted in Table 2.

**Table 2:** Table depicting chromogenic bacteria isolated from the studies included

Study	Organisms isolated	Characteristics of the organism
Mutsaddi S et al., 2018 (India)	A.naeslundii, A. actinomycetemcomitans, F. nucleatum	Gram positive rod shaped bacteria Gram negative facultative anaerobic; non motile Gram negative anaerobic bacteria
Gayatri A et al., 2017 (Indonesia)	Actinomyces spp, Prevotella spp	Gram positive bacteria; rod shaped, filamentous Gram negative anaerobic; rod shaped
Zhang Y et al., 2022 (China)	Neisseria Lautropia, Haemophilus, Aggregatibacter	Gram negative, cocci Gram negative facultative anaerobic Gram negative pleomorphic cocco bacilli Gram negative facultative anaerobic; non motile
Zhang F et al., 2017 (China)	Actinomyces	Gram positive bacteria; rod shaped, filamentous
Veses V et al., 2020 (Spain)	Bacteroidetes, Actinobacteria	Gram negative anaerobic or aerobic rod shaped Gram positive
Chen L et al., 2019 (China)	Firmicutes, Fusobacteria, Proteobacteria, Bacteroidetes, Actinobacteria, Candidate_division_TM7	Gram positive obligate anaerobic; cocci or bacilli Gram negative anaerobic bacteria Gram negative Gram negative anaerobic or aerobic rod shaped Gram positive Gram-positive
Çelik ZC et al., 2021 (Turkey)	Aggregatibacter, Bergeriella, Brachymonas, Capnocytophaga, Corynebacterium, Neisseria Rothia Streptococcus	Gram negative facultative anaerobic; non motile Gram-negative bacterium, Obligate aerobe Gram-negative, aerobic bacteria Gram-negative bacteria Gram-positive bacteria; aerobic Gram negative, cocci Gram-positive, aerobic, rod-shaped and non-motile Gram-positive coccus
Yanhui L et al., 2016 (China)	Leptotrichia Fusobacterium Streptococcus Mogibacterium Clostridiaceae, Peptostreptococcus	Non-motile facultative anaerobic/anaerobic bacteria Gram negative anaerobic bacteria Gram-positive coccus Gram-positive, strictly anaerobic Gram-positive, obligate anaerobic organisms Gram-positive, anaerobic cocci
Heinrich-Weltzien R et al., 2014 (Germany)	S. mutans, S. sobrinus, Lactobacillus sp., A. naeslundii, P. gingivalis, A. actinomycetem, P. intermedia, F. nucleatum	Gram-positive coccus Gram-positive coccus gram-positive, aerotolerant anaerobes or microaerophilic, rod-shaped, non-spore-forming bacteria Gram positive rod shaped bacteria Gram-negative, nonmotile, rod-shaped, anaerobic Gram negative facultative anaerobic; non motile Gram-negative, obligate anaerobic Gram negative anaerobic bacteria

## Discussion

The staining of teeth is a commonly encountered complaint in clinical practice. The causes of staining of teeth may be due to intrinsic or extrinsic causes. The dental pellicle that is formed on the surfaces of the teeth attracts a variety of external agents like chemicals, food substances and chromogenic bacteria resulting in staining of teeth [1]. The extent of adherence of these substances determines the color and composition of the staining [2]. The various reported color of the staining of teeth are black, brown, green, orange and staining occurring due to tobacco, mouthwash, heavy metallic salts. Chromogenic bacteria are unique organisms which can produce dental dyschromia (tooth discoloration) due to their capacity to produce color. There are typical organisms which are implicated in the causation of staining teeth due to these bacteria. There are various proposed mechanisms by which these organisms can cause staining [2, 28].

Prior to the 20th century, academics hypothesized that filamentous bacteria or Actinomyces were linked to the creation of black stain, based on the utilization of electron microscopy and in vitro culture technologies [28]. The next generation sequencing technologies has revealed the presence of a unique micro-ecological habitat in these stains and highlighted the concept of a core microbiome rather than one or two individual agents [29]. However majority of the studies have reported the presence of actinomyces genus in this core microbiome. Actinomyces are a type of bacteria that is Gram-positive, facultative anaerobic bacillus and it is a component of the dental plaque. Currently, a total of thirty three species have been recognized within this particular genus [20]. One of the causes was the ability of certain A. spp. to generate hydrogen suphide, particularly Actinomyces naeslundii, which could result in the creation of ferric sulphide [16]. Another factor was that Actinomyces has the ability to synthesize several siderophores including hydroxamic acid siderophores and catechol siderophore that exhibited a strong affinity for ferric ions (Fe^3+^ ions) [30–32]. In addition, few studies have also identified Rothia a member of the Actinomycetaceae family, which consists of filamentous, gram-positive, facultative anaerobic cocci to be an important constituent of core microbiome of the black stain [33].

Another genus which was reported by few studies to be associated with stain is Gram-negative cell wall structure. Neisseria is commonly found as a pair of cocci (diplococcus) and is a part of the normal microbiota of the host. This genus comprises 31 bacterial species, including Neisseria meningitidis and Neisseria gonorrhoeae, both of which can synthesize cysteine. Hydrogen sulfide (H_2_S) can be generated by cysteine metabolism, suggesting a potential association between Neisseria and the staining [34].

Apart from these two, several studies have highlighted the involvement of bacteria belonging to Prevotella that has the ability to create pigments that can vary in color from dark brown to black. Prevotella intermedia and Prevotella nigrescens rely on the heme component of hemoglobin as an essential source of iron for their bacterial development. P. nigrescens possess distinct surface proteins that can attach to hemoglobin. On the other hand, P. intermedia breaks down the hemoglobin to release the heme component onto the surface of the hemoglobin molecule. This process allows P. intermedia to then capture and utilize iron for its own growth [35, 36]. P. nigrescens and P. intermedia enzymatically break down oxyhaemoglobin to produce an intermediate compound called iron (III) protoporphyrin IX (Fe(III)PPIX)2O. This intermediate is then transformed into iron (III) protoporphyrin IX complex [Fe(III)PPIX.OH] through a decrease in pH. The acidic environment promotes the formation of insoluble complex on the cell surface [35, 37].

Research has demonstrated that Leptotrichia has the capability to generate hydrogen sulfide (H_2_S), a compound known for its distinct odor. Fusobacterium species have the ability to form aggregates with other microorganisms [38]. Chromogenic bacteria, specifically Porphyromonas gingivalis is also implicated to contribute to further effects [20, 39].

Nevertheless, there is ongoing debate over the core microbiome of black stain as a result of variations in methodologies and experimental designs of the studies conducted till now. The microbial etiology of black stains remains uncertain due to the limitations of existing research methodologies, which can only identify the microbial flora at the genus level. Furthermore, the absence of empirical verification at the level of individual species and the intricate composition of the black stain components have further impeded advancements in understanding pathogenic pathways [5].

The ambiguous origin of black stains complicates the identification of elements linked to its development. Only a small number of authors made efforts to identify connections between sex, age, diet, dental hygiene, socioeconomic position, medications, and the prevalence of BS. A recent study reported statistically significant associations were observed between tooth black stain consumption of water with high iron content, consumption of water with high pH, and having a high salivary pH [6]. Smoking, iron supplement intake, consumption of caffeinated drinks were not identified as risk factors for the occurrence of black stain. Consuming vegetables, fruits, dairy products, eggs, and soy sauce contributes to the formation of beneficial bacteria in the gastrointestinal tract [16, 25]. Consuming tap water, as opposed to bottled mineral or natural well water, appears to be linked to a greater occurrence of black stains in Brazil [40]. There is contradictory information about the impact of oral hygiene on black stain [5].

The clinical presentation of chromogenic staining of teeth differs from the stains caused by other agents. These stains are typically noted along the cervical margins of teeth in the form of confluent black dots. There are proposed classification of black staining of teeth as stated by Shourie et al. [41](1947), Koch et al. [42] (2001) and Gasparetto et al. [43] (2003).

**Fig. 3:**
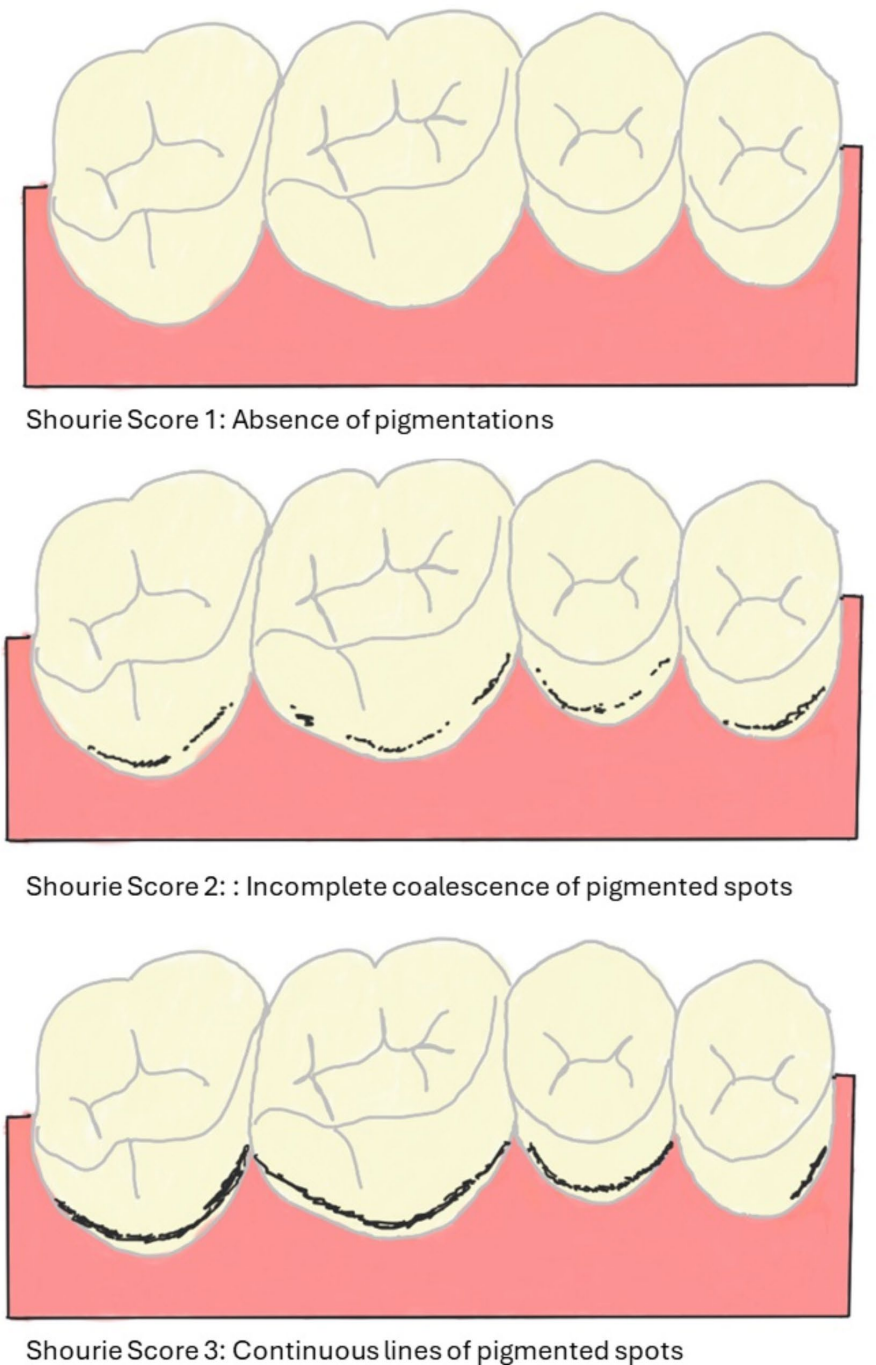
Pictorial representation of classification of chromogenic staining of teeth as proposed by Shourie et al. (1947)

Shourie (1947) (Fig. 3) developed a clinical scoring system for black staining of teeth as absence of pigmentations, incomplete coalescence of pigmented spots, continuous lines of pigmented spots scored 1,2 and 3 respectively [41]. Later, Koch et al. in 2001 modified this scoring system by including an additional criterion of presence of linear pigmented spots parallel to the gingival margin in at least two teeth with no evidence of cavitation [42].

**Fig. 4:**
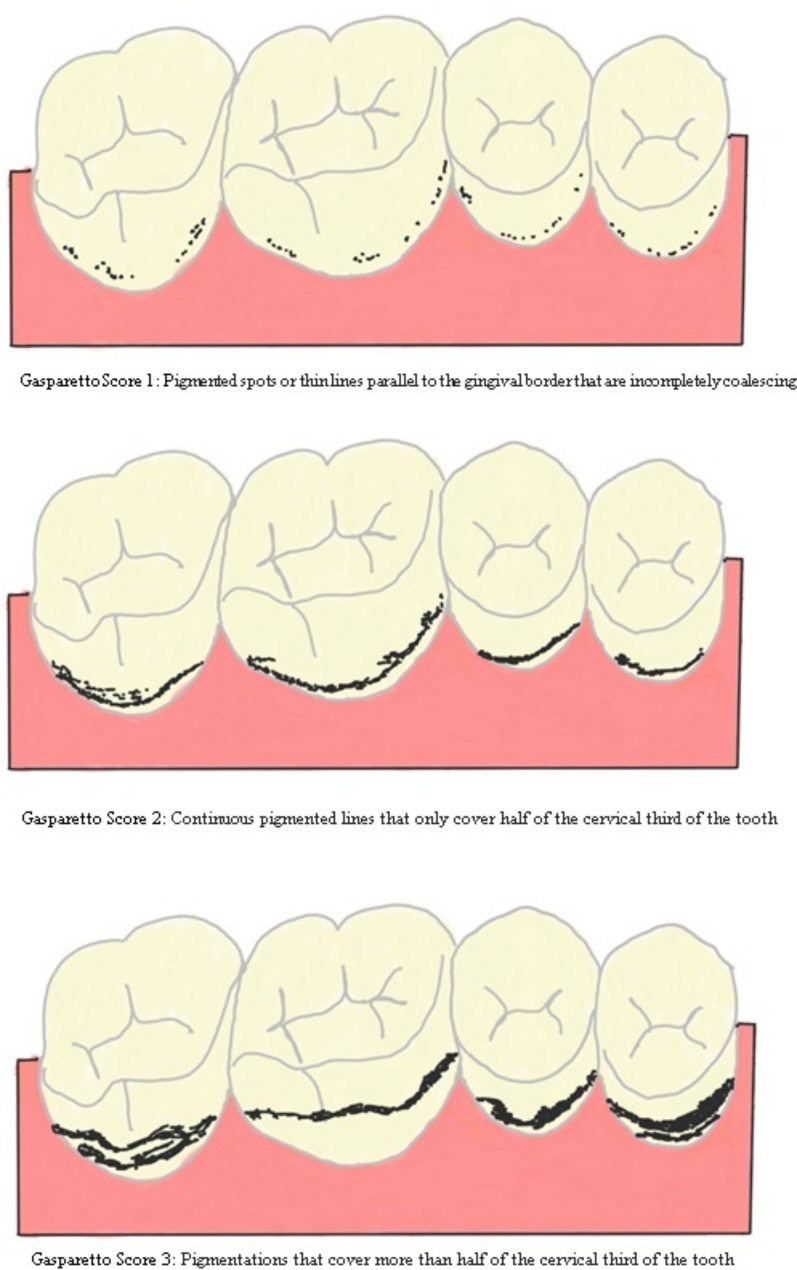
Pictorial representation of classification of chromogenic staining of teeth as proposed by Gasparetto et al. (2003)

Gasparetto et al. (2003) (Fig. 4) modified this classification as score 1 being pigmented spots or thin lines parallel to the gingival border that are incompletely coalescing; score 2 denoting continuous pigmented lines that only cover half of the cervical third of the tooth; and score 3 showing pigmentations that cover more than half of the cervical third of the tooth [43].

There is a variation in the prevalence of chromogenic staining reported in different populations and in primary mixed dentition. No studies have reported the prevalence in permanent dentition. Studies have reported a prevalence of 6.1–12.4% in primary dentition [7, 12, 15, 16, 20, 25, 26]. The estimation range of prevalence of chromogenic staining of teeth reported in mixed dentition is 1.5–18.5% [13, 14, 17, 18, 23, 24].

Staining of teeth often pose aesthetic concern for the patients and difficult to self-treat as they are not removed by routine toothbrushing and oral hygiene practices. The management goals are to restore aesthetics and ensure healthy gingival status in such cases. These cases are traditionally treated by scaling and polishing the teeth with fine pumice. Stains that are found resistant to oral prophylaxis and polishing are managed by mixing 3% hydrogen peroxide with pumice or using spray containing bicarbonate solutions [3, 44]. However, the most effective management of stubborn stains is by using jet prophylactic sprays containing mild abrasives. Chromogenic staining of teeth tend to recur within few months and the exact cause for the reappearance is poorly understood and warrants repeated scaling and polishing [8, 10, 11]. There are no specific management strategies researched and reported in literature addressing chromogenic staining of teeth. The challenge in the management lies due to the reappearance of the staining within a few months due to the recalcitrant nature of these stains to the conventional treatment modalities.

This scoping review revealed that the studies exhibited significant variability in clinical outcomes, microbiological parameters, and management approaches, resulting in inconclusive findings. Consequently, there is inconsistent data regarding the prevalence of chromogenic staining in primary and mixed dentitions. These results highlight the necessity for further research to evaluate diverse clinical, microbiological, and management strategies for chromogenic staining.

## Conclusion

This scoping review reveals the existing literature regarding the etiological, clinical characteristics and management of chromogenic staining of teeth. The prevalence of these stains reported in primary, mixed and permanent dentition is sparsely reported. Though the role of peculiar oral microbiota is well established, evidence regarding the management strategies to combat these recalcitrant staining remains a strong research question. This emphasizes the need for further research on chromogenic bacterial staining.

## Electronic supplementary material

Below is the link to the electronic supplementary material.

Supplementary Material 1

## Data Availability

The dataset supporting the conclusions of this article is included within the article. However, additional information can be requested from the corresponding author upon reasonable inquiry.

## Declarations

### Protocol and registration

The present review adhered to the guideline outlined in PRISMA and PRISMA-P checklist. The review protocol is registered at the International Prospective register of Systematic Reviews (PROSPERO) database under the protocol number CRD42024565446.
