# Supplementary material for: Chromogenic bacterial staining of teeth: a scoping review
Source: BMC Oral Health. 2025 Jan 11;25:55. doi: 10.1186/s12903-025-05441-4 (PMC11725193; doi:10.1186/s12903-025-05441-4)
Supplement: Supplementary file 1 — Supplementary Material 1 [file 12903_2025_5441_MOESM1_ESM.docx]

| Search strategy:1 | Scopus | chromogenic AND bacteria* AND teeth AND staining |
| --- | --- | --- |
|  | Medline | chromogenic AND bacteria* AND teeth AND staining |
|  | Cinahl | chromogenic AND bacteria* AND teeth AND staining |
|  | Embase | chromogenic AND bacteria* AND teeth AND staining |
|  | ProQuest | chromogenic AND bacteria* AND teeth AND staining |
| Search strategy:2 | Scopus | chromogenic AND stains AND teeth* |
|  | Medline | chromogenic AND stains AND teeth* |
|  | Cinahl | chromogenic AND stains AND teeth* |
|  | Embase | chromogenic AND stains AND teeth* |
|  | ProQuest | chromogenic AND stains AND teeth* |
| Filters applied for all the databases for exclusion | - Reviews - Letters to editors - Commentary - Book chapters/Books - Non-English language literature - Animal studies   **No time and country filters applied* | |
